# Notch3 signaling promotes tumor cell adhesion and progression in a murine epithelial ovarian cancer model

**DOI:** 10.1371/journal.pone.0233962

**Published:** 2020-06-11

**Authors:** Jessica C. Price, Elham Azizi, L. A. Naiche, Jenny G. Parvani, Priyanka Shukla, Seoyeon Kim, Jill K. Slack-Davis, Dana Pe’er, Jan K. Kitajewski

**Affiliations:** 1 Integrated Program in Cellular Molecular and Biomedical Studies, Columbia University, New York, NY, United States of America; 2 Program for Computational and Systems Biology, Parker Institute for Cancer Immunotherapy, Sloan Kettering Institute, Memorial Sloan Kettering Cancer Center, New York, NY, United States of America; 3 Department of Physiology and Biophysics, University of Illinois Chicago, Chicago, IL, United States of America; 4 Department of Microbiology, Immunology, and Cancer Biology, University of Virginia, Charlottesville, VA, United States of America; Cleveland Clinic Lerner Research Institute, UNITED STATES

## Abstract

High grade serous ovarian cancer (HGSC) is the most common and deadly type of ovarian cancer, largely due to difficulties in early diagnosis and rapid metastasis throughout the peritoneal cavity. Previous studies have shown that expression of Notch3 correlates with worse prognosis and increased tumorigenic cell behaviors in HGSC. We investigated the mechanistic role of Notch3 in a model of metastatic ovarian cancer using the murine ovarian surface epithelial cell line, ID8 IP2. Notch3 was activated in ID8 IP2 cells via expression of the Notch3 intracellular domain (Notch3IC). Notch3IC ID8 IP2 cells injected intraperitoneally caused accelerated ascites and reduced survival compared to control ID8 IP2, particularly in early stages of disease. We interrogated downstream targets of Notch3IC in ID8 IP2 cells by RNA sequencing and found significant induction of genes that encode adhesion and extracellular matrix proteins. Notch3IC ID8 IP2 showed increased expression of ITGA1 mRNA and cell-surface protein. Notch3IC-mediated increase of ITGA1 was also seen in two human ovarian cancer cells. Notch3IC ID8 IP2 cells showed increased adhesion to collagens I and IV *in vitro*. We propose that Notch3 activation in ovarian cancer cells causes increased adherence to collagen-rich peritoneal surfaces. Thus, the correlation between increased Notch3 signaling and poor prognosis may be influenced by increased metastasis of HGSC via increased adherence of disseminating cells to new metastatic sites in the peritoneum.

## Introduction

Ovarian cancer is the deadliest gynecologic malignancy and expected to be the 5^th^ leading cause of cancer death in women in the United States in 2017 [1]. The most common subtype of ovarian cancer is high grade serous ovarian cancer (HGSC). Standard treatment for HGSC includes surgical cytoreduction and combination chemotherapy with platinum and taxane [2, 3]. HGSC recurs in over 80% of cases that were initially disseminated beyond the pelvis [2, 4]. Due to rapid dissemination and few or non-specific symptoms, approximately 70–75% of ovarian cancers are discovered after dissemination of disease [5, 6].

HGSC metastasizes either by direct invasion of adjacent organs or by dissemination in the peritoneal fluid to new sites on the peritoneal lining [5, 6]. The peritoneal lining consists of a single layer of mesothelial cells over collagen-rich extracellular matrix and other stroma, and ovarian cancer is thought to preferentially attach to sites where the mesothelial layer is disrupted and extracellular matrix is exposed [7]. Once tumor cells adhere, they further reduce the integrity of the peritoneal mesothelial layer by triggering mesothelial contraction, metalloprotease expression, and increased cytokine and growth factor signaling, leading to increased invasion, motility, and further growth of metastases [6, 8].

Ovarian cancers often cause dramatic increases in the amount of peritoneal fluid, called ascites, which can result from tumor cell blockage of lymph nodes and/or an increase in vascular leakage in the tumors or adjacent tissues of the peritoneum [5, 6]. Ascites accumulation can increase the peritoneal fluid from less than 20mL to volumes over 500mL and can cause considerable patient distress [6]. Ascites accumulation is correlated with worse prognosis [6, 9], possibly because ascites contains growth factors, extracellular matrix proteins, proteolytic enzymes, and inflammatory signals that support tumor growth and dissemination [6]. Increasing intraperitoneal pressure due to ascites may also directly induce metastatic epithelial to mesenchymal transition [10].

Ovarian tumors frequently show high levels of Notch3 signaling, particularly in HGSC cases and HGSC-derived cell lines. Physiologic Notch3 signaling depends on Notch ligand interactions (Jagged or Delta-like) which leads to release of the Notch3 intracellular domain (N3IC) and subsequent formation of a transcriptionally active N3IC complex that includes both MAML and *RBPJκ* [11, 12]. Unbiased screening of almost 500 HGSC tumors demonstrated that the Notch3 pathway was altered in 22% of HGSC samples assessed [13] and patients with *NOTCH3* alterations demonstrated poor survival [14]. Most of the identified alterations were predicted to activate signaling, including copy number amplification, predicted activating mutations, and upregulation of Notch3 mRNA expression. The most frequent alterations of the Notch pathway were in *NOTCH3* itself, but other components of the Notch pathway were altered at lower frequency, including the *JAGGED1 and JAGGED2* ligand genes and Notch activation complex genes *MAML1*,*MAML2*, *MAML3*, and *RBPJκ* (*CSL*) [13]. Re-analysis of this dataset with additional tumors showed that over half of HGSC samples harbor deletions of *WWP2*, which targets NOTCH3 (but no other Notch genes) for endocytosis and degradation, and these *WWP2* mutations correlate with increased Notch3 expression [15]. Studies on independent sets of serous ovarian tumor samples have confirmed increased *NOTCH3* copy number, increased *NOTCH3* transcript levels, and increased NOTCH3 protein levels [16–21]. Patients with high levels of NOTCH3 showed shorter overall survival, higher grade and stage tumors, increased ascites accumulation, and increased recurrence [20, 21].

Notch3 expression levels also correlate with tumorigenic phenotypes, including proliferation, viability, cell cycle arrest, and apoptosis, in ovarian cancer cell lines *in vitro* indicating a role in tumor growth [14, 18, 22, 23]. Notch3 inhibition similarly reduced proliferation and induced apoptosis, indicating that Notch3 doesn’t just induce growth, but is a critical cell survival factor in some ovarian cancers. Notch3 signaling upregulates other tumorigenic behaviors, such as epithelial to mesenchymal cell transition and resistance to anoikis [14, 24, 25].

Despite the strong evidence implicating NOTCH3 in ovarian cancer development, the role of Notch3 signaling in HGSC dissemination and progression is not well understood. Here we demonstrate that in an *in vivo* mouse model of disseminated ovarian cancer, Notch3 activity decreases survival, upregulates expression of adhesion genes, and increases tumor cell affinity for extracellular matrix in the peritoneal wall.

## Results

### The ID8 IP2 murine model replicates some aspects of human HGSC

The ID8 cell line is commonly used to model ovarian cancer in mice and has similar clinical characteristics to HGSC, including morphologic features, tumor distribution, and ascites accumulation [26, 27], although it does not carry mutations characteristic of human HGSC [28]. The ID8 IP2 subline, generated by passing ID8 tumor cells twice through C57BL/6 mice *in vivo* [29], produces similar ascites and disseminated tumor nodules as do ID8 and human clinical disease (S2A–S2C Fig). The ID8 IP2 subline also develops tumors more rapidly *in vivo* than ID8; intraperitoneal injection of 5x10^6^ cells of the original ID8 line produced ascites accumulation and IVIS-visible tumors in approximately 16 weeks (114 days) [26], whereas tumor from 1x10^6^ cells ID8 IP2 line produced similar tumor criteria after 6–8 weeks [29]. ID8 derived tumors closely mimic HGSC histology, including papillary histology with high nucleus to cytoplasm ratio, prominent nucleoli and coarse chromatin clumping, cytoplasmic vacuolization and increased mitoses [27, 30]. When injected intraperitoneally into athymic nude mice, ID8 IP2 cells produce broadly disseminated densely nucleated papillary tumor nodules attached to the peritoneal wall (S2D and S2E Fig).

### Upregulation of Notch3 expression and signaling in ID8 IP2 cells

**Fig 1:**
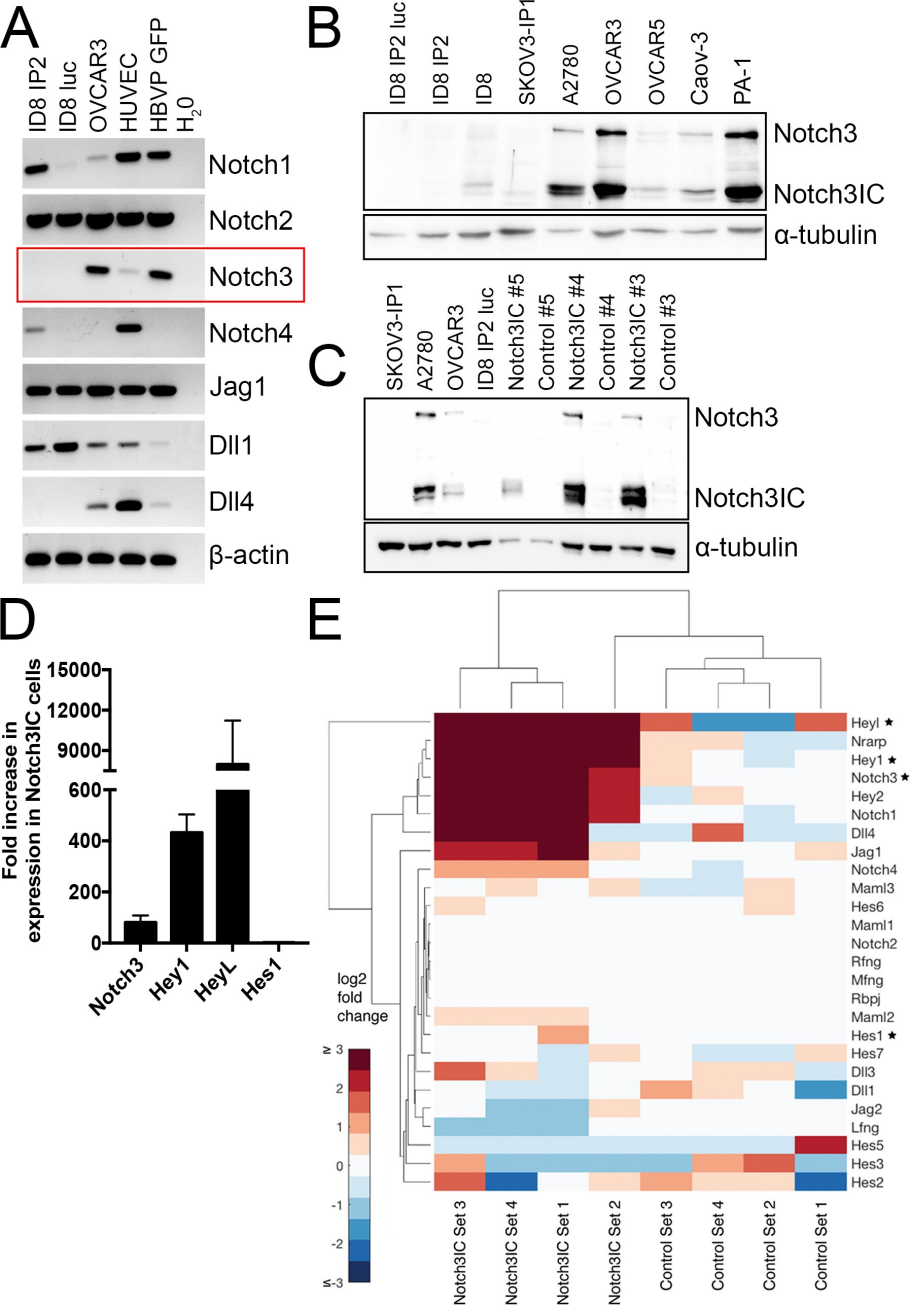
Ectopic expression of Notch3IC activates Notch 3 signaling in ID8 IP2 cells. (A) No Notch3 transcripts are detected in ID8 IP2 by semiquantitative RT-PCR (box). Notch receptors 1, 2, and 4 as well as ligands Jagged1 and Delta-like1 are detected in ID8 IP2 cells. (B) ID8 IP2 cells have undetectable levels of Notch3 protein when compared to cell lines previously characterized by detectable levels of Notch3 (OVCAR3, A2780, Caov3, and PA-1, Western blot). Uncropped images of all blots and gels are available in S1 Fig. (C) Representative Western blots show that expression of Notch3 intracellular domain is upregulated in Notch3IC lentivirally infected ID8 IP2 cell lines. Numbers indicate set number. (D) qRT-PCR indicates that Notch target genes are upregulated in Notch3IC Sets #1-#5 compared to matched Controls (error bars = S.E.M in all figures). This panel shows evidence of Notch activation across all 5 matched Sets, however, some variability was observed between sets (S3A Fig). (E) Selected established Notch target genes are upregulated in RNA-Seq data, demonstrating upregulation of active Notch3 signaling. Starred genes are also examined by qRT-PCR in panel D and S3A Fig.

To determine the baseline levels of Notch3 expression in the ID8 IP2 model, we assessed the mRNA levels associated with Notch receptor and ligand expression in the ID8 IP2 cell line and sublines we had stably transfected to express luciferase (ID8 IP2 luc). We found little to no expression of Notch3 mRNA or protein in ID8 IP2 or sublines, establishing the lack of Notch3 in this line (Fig 1A and 1B). We determined that the receptors Notch1, Notch2, Notch4, and Notch ligands Jagged1 and Delta-like1 are also expressed in ID8 IP2 (Fig 1A). However, Notch3 appears to have a unique role in HGSC [13, 16–19] based upon the alterations found in Notch3 levels in human HGSC.

We considered ID8 IP2 cells as a model with little evidence of Notch3 signal activation, and thus appropriate for determining the contribution of upregulated Notch3 signaling to ovarian cancer development. In other tumor types, amplification of Notch3 drives Notch3 signaling activity independent of ligand activation [31, 32]. To recapitulate this process, we engineered ID8 IP2 cells to express the Notch3 intracellular domain (Notch3IC), which constitutively drives Notch3 signaling without requirement for ligand binding or receptor cleavage [33]. ID8 IP2 cells were stably infected with lentivirus expressing Notch3IC and an IRES GFP or lentivirus control [12, 33]. A total of 5 independent lentiviral infections were completed to generate matched sets of ID8 IP2 Notch3IC and control (Set #1-#5). Increased expression of the Notch3 intracellular domain in the Notch3IC cells compared to matched control cells was verified by Western blot in all sets (Fig 1C). We confirmed activation of the Notch3 signaling pathway by assessing known direct transcriptional Notch targets Hey1 and HeyL via quantitative RT-PCR, which revealed dramatic upregulation of Notch signaling (Fig 1D). Previous data shows that Notch3, unlike other Notch genes, does not significantly activate Hes1 [33, 34]. No change was observed in the levels of Hes1 in the Notch3IC lines, confirming previous studies and suggesting that Notch3 is the primary source of Notch pathway activation in these cells. When mRNA from Sets #1-#4 were subjected to RNA-sequencing, Notch3 target genes Hey1, HeyL, and Nrarp showed strong upregulation in Notch3IC cells, again confirming active Notch3 signaling (Fig 1F). We also observed an increase in expression in certain other Notch receptor and ligand genes such as Notch1, Jagged1, and Delta-like4 (Fig 1E).

### Notch3 activity in tumors reduces survival and accelerates disease burden *in vivo*

Previous literature has suggested that Notch3 expression affects ovarian cancer by increasing survival, particularly anchorage-independent survival in peritoneal fluid [14, 18]. However, we did not see any significant difference in the number of viable cells after 48 hours of proliferation or anchorage-independent colony formation between Notch3IC and Control lines (S3B and S3C Fig). These data suggest that Notch3 signal activation in ID8 IP2 cells is not sufficient to substantially increase tumor cell numbers *in vitro*.

We investigated the effects of Notch3IC cells *in vivo* by intraperitoneally injecting NCR nu/nu mice with 2x10^6^ Notch3IC or Control cells from Set #1 to determine their rate of tumor formation and progression. We examined tumor burden via weekly IVIS imaging of luciferin luminescence. A small number of animals were removed from the study due to failure of tumor take, which occurred in similar numbers in Notch3IC and Control lines. Disease progression was assessed by measuring ascites accumulation, determined by weekly comparisons of each mouse’s abdominal circumference with its average circumference during the first 4 weeks of the study [27]. Mice were sacrificed when they reached humane endpoints, defined as a 25% increase in abdominal circumference or cachexia as assessed by body condition score [35].

**Fig 2:**
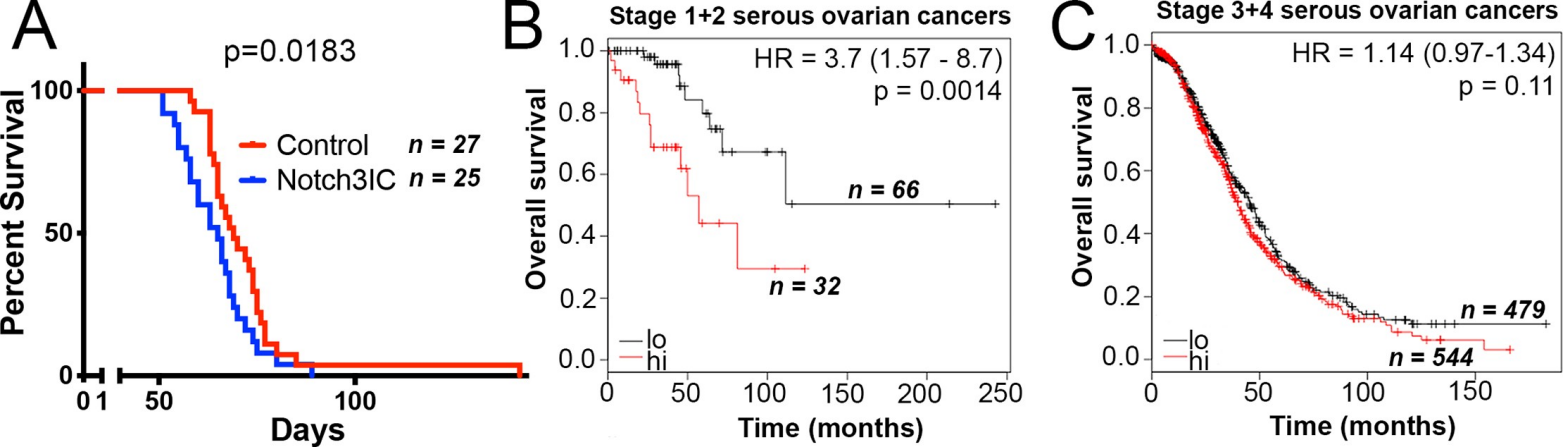
Notch3 activation correlates with reduced survival in early tumorigenesis. (A) Kaplan Meier survival curve demonstrates a significant decrease in survival time for mice implanted with Notch3IC cells using a Gehan-Breslow-Wilcoxon test (p = 0.0183), which is more sensitive to disproportionate early differences than the Mantel-Cox test (p = 0.0592). (B-C) Survival analysis at different stages of serous ovarian cancers using kmplot (http://www.kmplot.com/ovar/) shows highly significant correlation between high Notch3 expression and reduced survival in early stage ovarian cancers (B), but not late stage ovarian cancers (C).

A total of 25 mice injected with Notch3IC cells and 27 mice injected with matched Control cells were observed until humane endpoints were reached. The median survival of Notch3IC mice was 4 days shorter than Control mice (65 vs. 69 days). Although no significant difference in survival was observed with a Mantel-Cox assessment (p = 0.0592), Notch3IC tumor bearing mice displayed reduced survival using a Gehan-Breslow-Wilcoxon test (p = 0.0183, Fig 2A). The Gehan-Breslow-Wilcoxon test does not assume a consistent hazard ratio across the experiment and is better able to detect differences at earlier time points. The discrepancy between these tests suggests that there is a greater difference between groups in early deaths [36, 37]. Consistent with the hypothesis that Notch3 activation promotes early phases of metastatic disease, analysis of a cohort of human serous ovarian cancers revealed that high expression of Notch3IC predicts poor prognosis in stage 1 and stage 2 disease (p = 0.0014; Fig 2B), but not in stage 3 and stage 4 disease (p = 0.11; Fig 2C).

**Fig 3:**
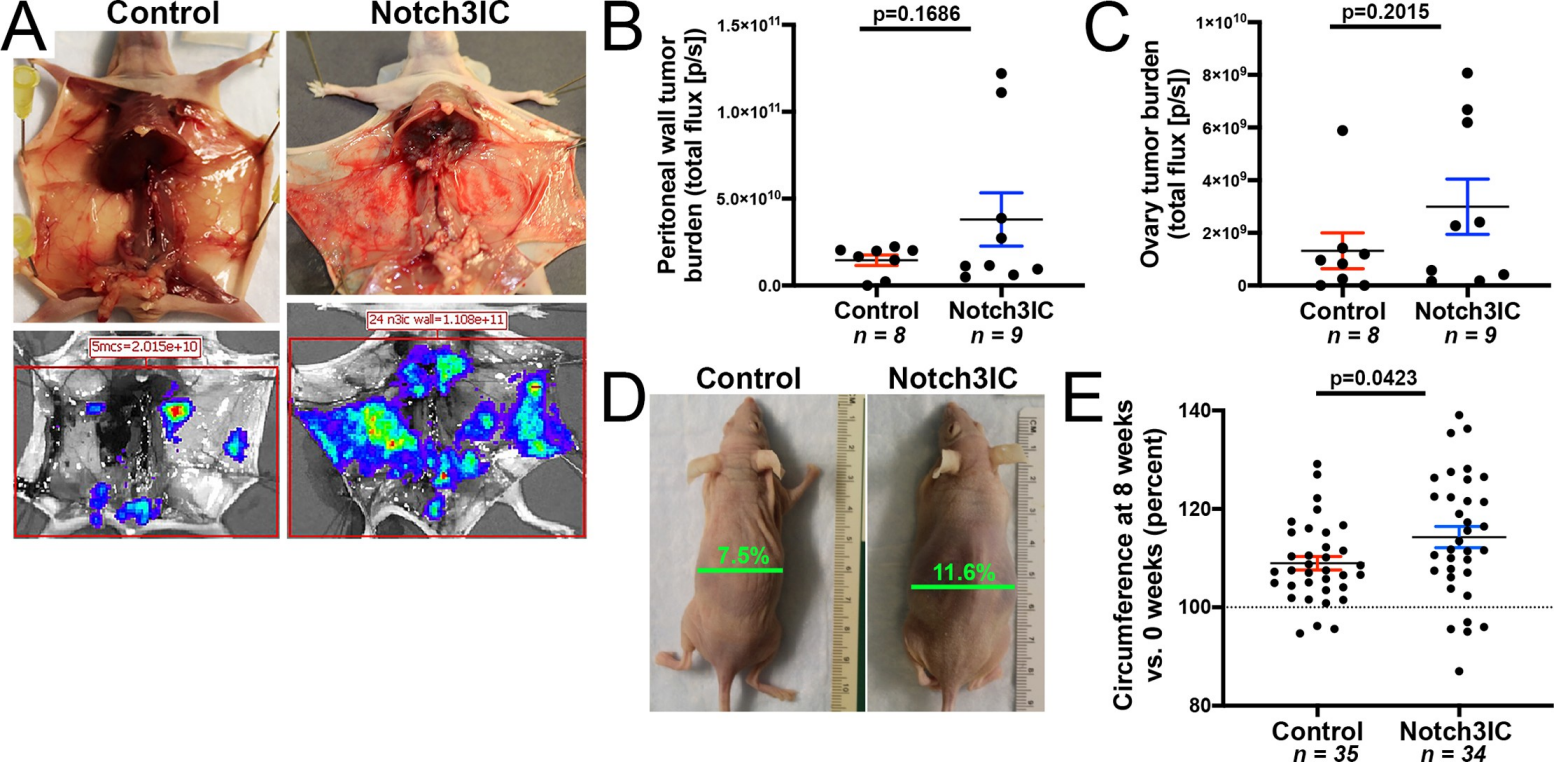
Notch3IC tumor bearing mice have increased ascites accumulation. (A) Representative images of the exposed peritoneal wall (top) and bioluminescent IVIS imaging (bottom) of Control and NotchIC tumor-bearing mice. (B-C) Notch3IC tumor bearing mice show non-significant increases in tumor burden in the peritoneal wall (B) and right ovary (C) as measured by IVIS signal at 8 weeks post implantation (p = 0.1686 and p = 0.2015, respectively, Welch’s t-test). (D) Representative photographs of Control and Notch3IC implanted mice at 8 weeks post implantation, with indicated circumference percent changes relative to circumference at start of experiment. (E) Notch3IC tumor bearing mice show a significant increase in circumference as a result of ascites accumulation at the 8-week time point (p = 0.0423 Welch’s t-test).

The earliest time of sacrifice due to ascites accumulation for this cohort of mice was 8 weeks post-implantation. We therefore implanted a set of ID8 IP2 cells (Set #2) into a second cohort of 17 mice (9 Notch3IC, 8 Control) to examine tumor burden specifically at this early timepoint. At 8 weeks post-implantation, we sacrificed this cohort, removed the abdominal organs, and assessed the tumor burden. Metastases were disseminated throughout the peritoneal cavity with individual animals exhibiting variable proportion of tumor burden on different organs. All tumor-bearing animals showed abundant lesions on the peritoneal wall and on the ovaries, and therefore these tissues were chosen as representative and imaged via IVIS. Bioluminescent signal averaged higher in Notch3IC tumor bearing mice, however, there was no significant difference between the tumor burden on the peritoneal wall or right ovary and uterine horn in this smaller set of mice (p = 0.1686, p = 0.2015 respectively, Fig 3A–3C).

All mice (totaling 34 Notch3IC and 35 Control) were assessed for ascites accumulation, a measure of disease progression, by measuring circumference at 8 weeks after tumor injection and comparing to baseline circumference. Notch3IC mice displayed a significantly greater percent increase in circumference at 8 weeks (14.3% ± 2.2% [standard error of mean] increase vs. 8.9% ± 1.4% increase, p = 0.0423, Fig 3D and 3E). Notch3IC tumor bearing mice also showed significantly greater absolute increase in circumference (1.08 ± 0.15 cm vs. 0.69 ± 0.10 cm, p = 0.0386, Welch’s t-test). Taken together, these data show that Notch3 signal activation in this model of ovarian cancer reduces survival and leads to an increase in ascites accumulation without substantially increasing tumor cell proliferation.

### Notch3 activity positively regulates pathways associated with adhesion and extracellular matrix in ID8 IP2 cells

To determine the mechanism by which Notch3 promotes metastasis, we examined the gene specific expression changes caused by Notch3 signal activation by whole-genome mRNA sequencing (RNA-Seq) of the ID8 IP2 Notch3IC cells compared to Controls (Sets #1-#4). 478 genes were significantly upregulated and 163 significantly downregulated with an adjusted p value (p_adj_) < 0.1 and average log2 fold change ≥ |1|. The complete RNA sequencing dataset is available at accession GSE132737 in the NCBI Gene Expression Omnibus repository at https://www.ncbi.nlm.nih.gov/geo/query/acc.cgi?acc=GSE132737. These significantly regulated genes were interrogated for pathway enrichment using the Database for Annotation, Visualization and Integrated Discovery (DAVID) [38]. In 3 of the 4 databases probed by DAVID, the most significantly enriched pathways included those related to cell adhesion and the extracellular matrix (ECM) (Table 1). Other gene sets, such as angiogenesis and Akt signaling, were identified in a subset of probed databases, suggesting that these mechanisms may also contribute to Notch3 function. Independent analysis using the Gene Set Enrichment Analysis (GSEA) database [39, 40], which accounts for the magnitude of change and pathway weight of each gene, revealed strong enrichment in pathways related to adhesion and ECM, particularly collagen and integrin genes (Table 2).

**Table 1:** Whole-genome expression profiling of Notch3IC cells shows significant regulation of adhesion and ECM categories by DAVID analysis.

Pathway database	Gene Cluster	Gene Count	p Value	Fold Enrichment
GOTERM	**Cell adhesion**	43	5.07E-14	3.968
GOTERM	Multicellular organism development	56	1.75E-09	2.422
KEGG	**Focal adhesion**	21	1.65E-08	4.662
KEGG	**ECM-receptor interaction**	14	4.18E-08	7.311
GOTERM	Mesenchymal cell development	6	1.09E-07	38.366
GOTERM	Angiogenesis	22	1.14E-07	4.086
KEGG	PI3K-Akt signaling pathway	25	4.51E-07	3.273
GOTERM	Blood vessel development	12	4.53E-07	7.565
REACTOME	**Integrin cell surface interactions**	12	4.00E-06	5.979
REACTOME	**ECM proteoglycans**	9	5.44E-06	8.853
KEGG	Hypertrophic cardiomyopathy	10	4.70E-05	5.817
KEGG	Arrhythmogenic right ventricular cardiomyopathy (ARVC)	9	1.35E-04	5.825
REACTOME	**Collagen biosynthesis and modifying enzymes**	9	1.60E-04	5.660
REACTOME	cGMP effects	5	2.61E-04	14.755
REACTOME	** Syndecan interactions**	5	4.78E-04	12.788
BIOCARTA	Function of SLRP in Bone	3	6.84E-03	21.016
BIOCARTA	Role of Tob in T-cell activation	4	2.99E-02	5.604

Genes that were shown to be significantly upregulated in Notch3IC cells as compared to Control by RNA-sequencing were subjected to DAVID analysis. Among the 17 most significantly regulated gene clusters, 7 are related to adhesion or extracellular matrix (bold).

**Table 2:** Notch3IC cells show significant enrichment of adhesion and ECM gene sets in GSEA analysis.

Gene Sets	Gene Count	NOM p-val	Normalized Enrichment
NABA_COLLAGENS	29	2.37E-03	1.73
REACTOME_COLLAGEN_FORMATION	30	1.83E-02	1.66
PID_INTEGRIN1_PATHWAY	42	1.73E-02	1.62
ONDER_CDH1_TARGETS_2_UP	69	1.61E-02	1.61
NABA_BASEMENT_MEMBRANES	24	3.14E-02	1.58
PID_INTEGRIN_CS_PATHWAY	16	4.95E-02	1.54
REACTOME_INTEGRIN_CELL_SURFACE_INTERACTIONS	36	7.26E-02	1.47

Selected gene sets related to adhesion and ECM pathways were significantly regulated in Notch3IC cells by GSEA analysis.

**Fig 4:**
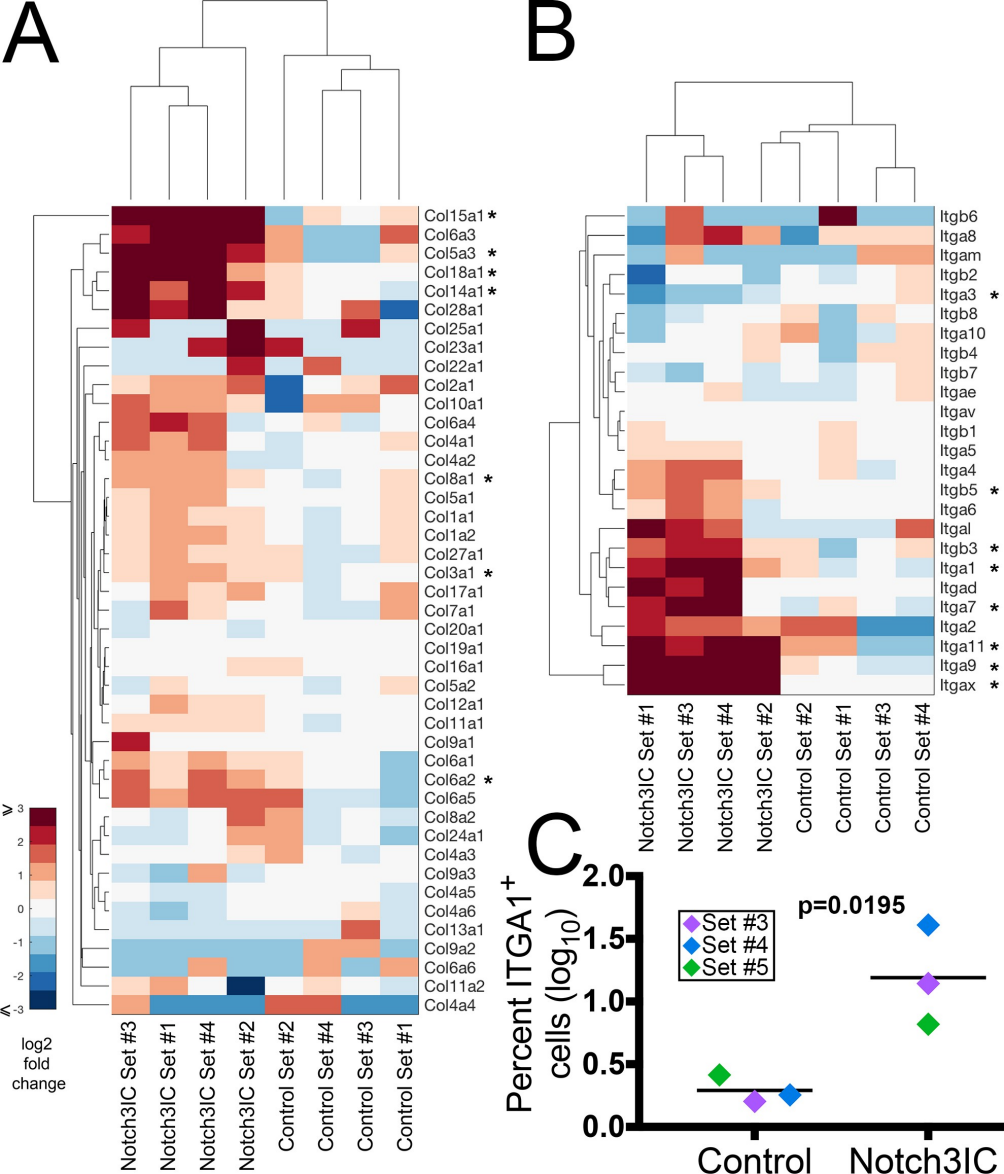
Expression of specific members of the collagen and integrin families are upregulated in Notch3IC cells. (A) Heat map of RNA-seq results of collagen gene family. Collagens marked by asterisks are significantly regulated (p ≤ 0.1, average log_2_ fold change ≥ 1) in Notch3IC cells. (B) Heat map of integrin gene family. Multiple integrins are upregulated in Notch3IC cells, while Itga3 is significantly downregulated. (C) ITGA1 is significantly upregulated on the surface of Notch3IC cells as assessed by flow cytometry (p = 0.0195, Student’s t-test, each Set assessed twice and results averaged).

Changes in cell adhesion and migration can profoundly affect metastasis, and a previous *in vitro* study suggested that Notch3 increases adhesion between ovarian tumor cells and co-cultured mesothelial cells [41]. Collagen and integrin genes have critical roles in attachment of ovarian tumor cells to the peritoneum and signaling in metastatic lesions [42, 43]. We therefore examined the gene signature in these adhesion and ECM pathways to identify specific candidates that could affect metastasis. Collagen genes *Col3a1*, *Col5a3*, *Col6a2*, *Col8a1*, *Col14a1*, *Col15a1*, and *Col18a1* were significantly upregulated (p_adj_ = 1.51E-02, 9.85E-03, 3.76E-02, 2.30E-02, 3.16E-09, and 4.12E-04 respectively) (Fig 4A). Significantly upregulated integrin genes include *Itga1*, *Itga7*, *Itga9*, *Itga11*, *Itgb3*, and *Itgb5* (p_adj_ = 7.70E-03, 2.30E-02, 5.80E-06, 1.32E-06, 6.51E-03, and 6.17E-03 respectively) (Fig 4B).

We further examined ITGA1, a significantly upregulated collagen receptor component that was identified as a common element in multiple upregulated pathways (log_2_ fold change 3.8, p_adj_ = 0.00770). The peritoneal ECM is rich in collagens I and IV, and invasive ovarian cancer cells preferentially bind collagen I [43]. ITGA1 dimerizes with ITGB1 to bind collagens I, IV, VI, and fibrillar collagens, making it an excellent candidate to affect tumor cell adhesion to the peritoneum [44]. The level of ITGB1 did not change significantly in Notch3IC cells compared to controls but was found at high levels in both Control and Notch3IC (DEseq baseMean Control 17,913.9, baseMean Notch3IC 19,414.9), suggesting abundant availability for dimerization with ITGA1. For integrins, increased transcript levels do not necessarily correlate with increased presentation of protein on the cell surface capable of influencing adhesion; we therefore directly analyzed ITGA1 levels by flow cytometry. We stained Sets #3–5 of Notch3IC and Control lines with antibodies against ITGA1 in two duplicate experiments and observed a significant increase in the percent of Notch3IC cells which were ITGA1 positive, with a roughly 10-fold average increase over the Control sets (p = 0.0195, Students’s t-test, Fig 4C and S4A–S4E Fig). No obvious difference was observed in the staining intensity of individual cells. These results indicate that increased RNA expression correlates to a larger number of Notch3IC cells that express cell-surface ITGA1. Similar experiments to confirm ITGA11 surface expression failed due to poor performance of antibody in flow cytometry tests.

We further inquired whether the increase in ITGA1 expression was a general feature of Notch signaling. More specifically, we asked if IGTA1 also responded to the increase in Notch1 observed in Notch3IC cells (Fig 1E). When we overexpressed the Notch1 intracellular domain (Notch1IC), we observed a significant increase in ITGA1 surface expression by flow cytometry (p = 0.0395, Welch’s t-test, S4F and S4G Fig). However, ITGA1 expression was only increased ~0.5 fold in response to Notch1IC, as opposed to ~10 fold in response to Notch3IC, suggesting that Notch3 is the critical regulator of ITGA1 in ID8 IP2 cells.

### Notch3IC positively regulates ITGA1 in human ovarian cell lines

To confirm that Notch3IC also increases ITGA1 expression in human ovarian cancers, we overexpressed Notch3IC in two late-stage human ovarian cancer cell lines, OVCA429 and OVSAHO (S5A Fig). Increased Notch3IC signaling in these cells was confirmed by RT-PCR assessment of Notch3IC target genes, Notch3 and HeyL (S5B Fig). Consistent with our findings in the ID8 IP2 cells, overexpression of Notch3IC in the OVCA429 and OVSAHO cells resulted in a greater than 10-fold increase in percentage of cells with surface expression of ITGA1 in both cell lines, although no replicates were performed within each cell line (S5C–S5H Fig). We did not observe changes in adhesion in these cell lines, possibly due to higher basal Notch3IC expression or differences in disease stage.

### Notch3 activity increases ID8 IP2 cell adhesion to collagen extracellular matrix

**Fig 5:**
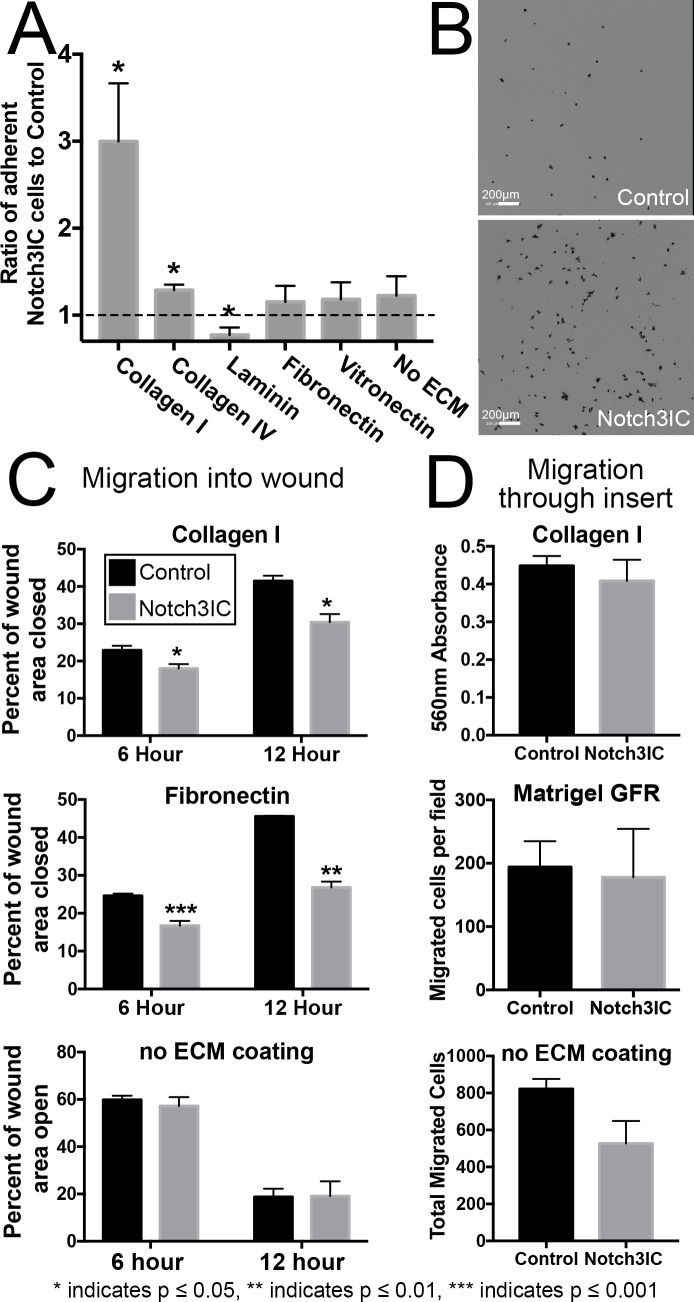
Notch3IC cells show increased adherence to collagens and decreased collective migration on ECM substrate, but unaltered invasion through ECM. (A) The ratio of Notch3IC cells to Control cells adhered to plates coated with indicated extracellular matrix. Dotted line indicates no change. Notch3IC cells display a significant increase in adherence to collagen I (p = 0.0118, Welch’s t-test) and collagen IV (p = 0.0163) and a small but significant reduction in adhesion to laminin (p = 0.0474). There is no significant difference in adhesion to fibronectin, vitronectin, or uncoated tissue culture dishes (p = 0.6237, p = 0.3075, p = 0.4206, Welch’s t-test). Sets #3–5 of ID8 IP2 were assessed in triplicate on 3 separate occasions. The Notch3IC mean was compared to the Control mean for each line. (B) Representative images of ID8 IP2 and Control cells adhered to collagen I-coated plates. (C) The ability of Notch3IC cells to collectively migrate into an open wound area is significantly reduced on collagen I (p = 0.0432, p = 0.0181 for 6 and 12 hours respectively) and fibronectin (p = 0.0004, p = 0.0020 for 6 and 12 hours respectively), but not on uncoated tissue culture dishes (p = 0.5422, p = 0.9637 for 6 and 12 hours respectively). Sets #3-#5 were assessed in quadruplicate wells twice. Graphs display the average of the duplicate experiments for each set. (D) The ability of Notch3IC cells to invade through insert pores coated with Collagen I, coated with Matrigel GFR, or uncoated was not significantly changed relative to Controls (p = 0.5634, p = 0.6076, and p = 0.1205, respectively). Sets #3-#5 of ID8 IP2 were assessed in triplicate wells and each set was averaged.

Based on the RNA-Seq upregulation of extracellular matrix ligand and receptor genes, as well as the confirmed upregulation of expression of surface ITGA1, we sought to determine if activated Notch3 causes ovarian tumor cells to interact differentially with different extracellular matrices present in the peritoneum. We therefore assessed the ability of Notch3IC and Control cell lines to attach to different extracellular matrices *in vitro*: fibronectin, laminin, vitronectin, collagen I, and collagen IV [43, 45]. Equal numbers of Notch3IC or Control cells were added to plates coated with each ECM protein, allowed to settle and attach for a specified period of time, and the number of cells which successfully attached to the purified ECM protein were counted. It was found that all ID8 IP2 cells bound well to vitronectin- and fibronectin-coated plates, and NOTCH3 ICD expression did not further enhance attachment (Fig 5A). Notch3IC tumor cells, however, were able to better adhere to collagen-coated plates than Control cells (Fig 5A and 5B). Attachment of Notch3IC cells to collagen I was increased by 3.1 ± 0.7 fold (p = 0.0118, Welch’s t-test) and increased to collagen IV by 1.3 ± 0.1 fold (p = 0.0163, Welch’s t-test). These results suggest that Notch3 activation in ovarian tumor cells may have increased attachment capabilities to the collagen-rich peritoneal wall.

### Notch3 activation reduces ECM-mediated migration but does not change invasion in ID8 IP2 cells

In some contexts, ovarian cancer cell binding to collagen causes increased migration and invasion, steps of peritoneal metastasis [42]. We therefore predicted that Notch3 activation in ID8 IP2 cells would likewise increase migratory and invasive behaviors on collagen substrates.

We investigated migration by growing Notch3IC and Control cells on collagen I coated Oris assay plates (Platypus Technologies). At confluence, the plug was removed to reveal a collagen I coated cell-free “wound” and the percent area of the wound covered by cells was measured over time. We assessed 3 ID8 IP2 sets (Sets #3-#5) in quadruplicate wells in 2 replicate experiments. Surprisingly, Notch3IC cells seeded on collagen I displayed a significant reduction in capacity to migrate across collagen I at both 6 and 12 hours (p = 0.0432, p = 0.0181, Welch’s t-test, Fig 5C). To determine if this effect was matrix-specific, we repeated these assays on fibronectin coated Oris assay plates, and also observed a significant reduction in migration of Notch3IC cells compared to Control cells at both 6 and 12 hours (p = 0.0004, p = 0.0020, Welch’s t-test, Fig 5C).

Conversely, when we assessed migration in the absence of extracellular matrix by creating a wound across a confluent monolayer of cells on an uncoated tissue culture plate, there was no significant difference between Notch3IC and Control cells in migration into the opened wound (p = 0.4142, p = 0.5422, p = 0.4133, p = 0.9637 for 3, 6, 9, and 12 hours after wound formation respectively, Welch’s t-test) (Fig 5C). These results suggest that general migratory ability is not changed by Notch3 activation, but Notch3 signaling reduces ECM-mediated migration.

In light of the findings demonstrating that adhesion to and migration over extracellular matrix are altered between Notch3IC and Control cells, we investigated whether Notch3 activation has an effect on invasion. We measured invasive capacity by quantifying cell migration through ECM-coated Boyden chambers, which requires invasion of the cells through the matrix to pass through a porous membrane [46]. A direct count of the number of cells which had invaded to the bottom surface of the membrane showed that there was no significant difference between Notch3IC and Control cells in invasion in Boyden chambers pre-coated with collagen I (p = 0.5634, Welch’s t-test), pre coated with Matrigel GFR, a laminin rich amalgam of ECM and tumor-derived components [47] (p = 0.8634), or uncoated Boyden chambers (p = 0.1205). These results suggest that post-attachment invasive activity is unchanged in ID8 IP2 cells with activated Notch3 (Fig 5D).

## Discussion

Expression of Notch3 and activation of its downstream pathway is strongly correlated with poor prognosis in ovarian cancer. Our data demonstrate that activating Notch3 signaling is sufficient to accelerate disease progression and increase ascites accumulation in a mouse model of ovarian cancer, ID8 IP2 in the peritoneal cavity.

Mortality in ovarian cancer is generally due to metastasis, where cancer cells and spheroids detach from the primary tumor [43, 48], distribute in the peritoneal fluid, and attach to new sites [48, 49]. Even when ovarian cancer cells spread via hematogenous dispersal, they most commonly metastasize to the peritoneum [50–52]. The surface of the peritoneum is comprised of collagen-rich ECM overlaid with a single layer of mesothelial cells, which secrete glycosaminoglycans, surfactant, and proteoglycans that prevent adhesion between the peritoneal organs and may form a poor attachment point for tumor cells [7, 43, 53]. Ovarian cancer cells therefore preferentially adhere to the ECM under the mesothelium, while mesothelial cells are inhibitory to tumor cell attachment [43, 45, 49, 53–55]. In the normal peritoneal environment, there are areas of exposed ECM at mesothelial intercellular junctions, omental immune aggregates, and highly vascularized areas, which may provide initial attachment points for ovarian cancer cells [7, 51]. As ovarian cancer progresses, further damage to the mesothelium by inflammatory signals allows for increased exposure of the ECM and therefore provides more chance of attachment for tumor cells [7, 54, 56]. Attachment to collagen I causes additional invasive behavior in ovarian tumor cells, including activation, migration, proliferation, and secretion of factors that remodel the ECM and further metastasis [54]. Our data in 3 different ovarian cancer cell lines consistently suggest that Notch3 activation leads to increased likelihood of tumor cell binding to new sites in the peritoneum. These changes may explain why Notch3 expression is strongly correlated with lethality in ovarian tumors that have been identified in early stages: Notch3 expression increases the likelihood of stage 1 and 2 cancers to colonize distant sites leading to lethal metastatic stages 3 and 4.

We observe a significant difference in ascites accumulation at an early 8-week timepoint and statistically significant reduced survival with Gehan-Breslow-Wilcoxon analysis that better detects early survival differences, which suggests that the role of Notch3 occurs in the early stages of tumorigenesis. We propose a novel model for Notch3 action in ovarian cancer where Notch3 activation leads to increased seeding of detached tumor cells to new sites in the peritoneum. While previous studies have shown that loss of Notch3 reduces cell survival in Notch3-overexpressing ovarian cancer [14, 18], our results show that acquisition of Notch3 is not sufficient to increase survival, proliferation, or anchorage independent growth in these cells. We demonstrate that in the mouse ID8 IP2 and human OVCA429/OVSAHO ovarian cancer cell lines, Notch3 signaling upregulates the collagen I receptor subunit, ITGA1. Elevated ITGA1 expression increases adhesion to collagens I and IV in the ID8 IP2 cells. These results suggest that Notch3 activation increases the likelihood of ID8 IP2 cells to adhere to new peritoneal sites with exposed collagen I, thus increasing the frequency of new metastatic colonies. Previous studies have shown that Notch transcriptional complexes can bind directly to the enhancer of IGTA1 and other integrin genes, suggesting that integrins are direct transcriptional targets of Notch signaling [57]. In contrast, the late-stage human ovarian cancer cell lines OVCA429 and OVSAHO, harbor basal Notch3IC expression and may not readily gain additional adhesive properties in response to increased Notch3 activation, and the loss of correlation between Notch3 and survival in human stage 3 and 4 serous ovarian cancers suggests that later stage cancers upregulate Notch3-independent adhesion mechanisms.

We focused here on Itga1 due to its ability to bind collagen I, the most prevalent component of the peritoneal ECM [43, 45, 53]. However, activation of Notch3 also upregulates other integrins capable of binding peritoneal ECM components such as Itga11, which binds collagens I and IV, Itga7, which binds laminin, Itga9, which binds fibronectin, and Itgb3 and Itgb5, which both form dimers that bind fibronectin and vitronectin [58]. The reduced migration observed on fibronectin, for example, hints at tumor cell-ECM interaction regulated by Itgb3 and/or Itgb5. Multiple integrins may contribute to Notch3-mediated enhanced adhesion and accelerated disease progression observed *in vitro* and *in vivo* and could be explored in future studies. It is also possible that there is synergism between Notch3-mediated and Notch3-independent changes in adhesion, in particular upregulation of *COL11A1*, which is unaltered by Notch3 signaling but associated with poor survival in human HGSC and ovarian tumor progression in mouse models [59].

The upregulation of integrin genes by Notch3 activation may also play a role in survival of detached tumor cells in the peritoneal fluid, which must evade detachment-induced apoptosis (anoikis) and lymphatic clearance. Ovarian tumor cells can form spheroids, which escape anoikis by mutual integrin/ECM signaling, cadherin signaling, and VEGF-A/VEGFR2 signaling [7, 43, 60]. Integrin signaling from spheroids also induce the exposure of additional ECM attachment sites between mesothelial cells [43]. Notch3 expression in early stage ovarian cancers may increase the likelihood of progression to metastatic later stage cancers. Investigation of Notch3-induced effects on ovarian spheroids is warranted in future studies.

Our results suggest that Notch3 plays an important role in the early stages of ovarian metastasis, and thus inhibition of Notch3 signaling may provide clinical benefit in prevention of progression of ovarian cancer from low-burden to high-volume dissemination. Unfortunately, clinically available general inhibitors of Notch signaling, such as γ-secretase inhibitors (GSIs), cause gastrointestinal toxicity and likely have off target effects [61, 62]. Antibodies against the Notch ligand DLL4 have been tested clinically against tumor angiogenesis, but are unlikely to block Notch3 signaling, which appears to be activated by the ligand JAG1 in ovarian cancers where Notch3 signaling has not become ligand-independent [41, 62–64]. JAG1 is expressed in the tumor microenvironment by peritoneal mesothelium and tumor-associated endothelium and loss of JAG1 in adjacent cells reduces tumor cell adhesion and growth, suggesting that microenvironmental activation of Notch3 is critical in tumor progression [41, 65]. Notch decoys that specifically block Jagged signaling have been developed and may merit testing for activity against certain ovarian cancers [11].

Our results strongly suggest that integrin dimer α1β1 is a key downstream factor in Notch3-induced metastasis, and targeting this complex may reduce metastasis in both Notch3-induced and Notch3-independent ovarian cancers. Currently, clinically available inhibitors target integrins αvβ3, αvβ5, α5β1, and the αv and α2 subunits of dimers [66, 67]. In vitro testing of anti-integrin β1 antibodies on ovarian cancer cells shows reduced spheroid formation, adhesion, and migration, supporting our results and suggesting that these antibodies may become clinically important [68, 69]. The naturally occurring collagen IV-derived molecule, arresten, is believed to function through integrin α1β1 inhibition and may show synergistic effects between its demonstrated anti-angiogenic properties and anti-adhesion activity in ovarian cancer [70].

Our examination of the role of Notch3 signaling highlights the importance of adhesion in metastasis, and suggests new targets for controlling ovarian cancers. Further study of other Notch3 targets may elucidate other important mechanisms of ovarian cancer progression.

## Materials and methods

### Cell culture

The mouse ID8 IP2 cell line was developed in the lab of co-author Jill Slack-Davis and provided directly to the Kitajewski lab [29]. ID8 IP2 cells were cultured in DMEM with 10% FBS, 1% ITS Liquid Media Supplement (Sigma-Aldrich I3146), and 1% penicillin-streptomycin, and infected with lentiviral vector FUW-luciferase-mCherry [71]. Human ovarian cancer cell lines OVCAR3. OVSAHO, and OVCA429 were a kind gift of Dr. Joanna Burdette (University of Illinois Chicago), A2780 was a kind gift of Dr. Tian-Li Wang (Johns Hopkins), and SKOV3-IP1 was a kind gift of Dr. Olga Razorenova (University of California Irvine). OVCAR3, A2780, OVSAHO and SKOV3-IP1 were cultured in RPMI 1640 with 10% FBS and 1% penicillin-streptomycin, while OVCA429 was cultured in MEM with 10%FBS, 1% L-glutamine, 1% NEAA, 1% Sodium pyruvate and 1% penicillin-streptomycin. Mouse and human cells were lentivirally infected with virus derived from a pCCL vector encoding an HA-tagged Notch3 intracellular domain (codons 1664–2318) followed by an IRES-GFP (Notch3IC, Fig 1C) or empty pCCL vector. Five matched sets of Notch3IC and control ID8 IP2 lines were generated, identified as Sets #1-#5 throughout. Human cells were freshly infected prior to each experiment. Post-hoc testing suggests that all ID8 IP2 lines were mycoplasma positive throughout these experiments.

### Immunoblotting

Western blot was performed on 15μg of cell lysates subjected to SDS-PAGE electrophoresis in an 8% gel and subsequently blotted to nitrocellulose membrane. Membranes were stained with primary antibodies anti-Notch3 at 1:1000 (Santa Cruz Biotechnology Inc., sc-5593), anti-Notch1 at 1:1000 (Cell Signaling #3608), or anti-α-Tubulin at 1:1000 (Sigma-Aldrich, T6074) and secondary peroxidase-conjugated antibodies goat anti-rabbit at 1:5000 (Sigma-Aldrich, A6154) and sheep anti-mouse at 1:5000 (GE Healthcare Life Sciences, NA931VS). Enhanced chemiluminescence was used to detect secondary antibodies (Thermo Scientific SuperSignal West Femto Chemiluminescent Substrate, or GE Healthcare Life Sciences Amersham ECL).

### RT-PCR

RNA was extracted from harvested cells using the RNeasy Mini Kit (QIAGEN, 74104), and cDNA was generated using Verso^™^ cDNA synthesis Kit (Thermo Scientific^™^, AB1453A). Semiquantitiatve RT-PCR was performed with 150ng of cDNA template and 1 unit of Maxima Hot Start Taq polymerase (ThermoFisher Scientific) for 35 cycles using primers indicated in S1 Table.

For qRT-PCR, cDNA for Notch3, Hes1, Hey1 and HeyL were assessed on a Life Technologies ABI ViiA7^TM^ Real-Time PCR system using ABsolute Blue qPCR SYBR Green ROX (Thermo Scientific, AB4163A) reagent and primers indicated in S1 Table. Mean threshold cycle numbers (Ct) were determined for each gene and compared to the mean Ct of beta actin. The fold change was calculated by comparing the Ct of Notch3IC and Control cell lines normalized to ß-actin Ct. Results were graphed in Graphpad Prism 7 software.

### ID8 IP2 *in vivo* modeling

All animal experiments were approved by The Columbia University Institutional Animal Care and Use Committee and comply with the USPHS Policy on Humane Care and Use of Laboratory Animals. All personnel working with mice in this study have attended the Columbia University Laboratory Animal Regulatory Lecture, passed courses TC0900 Introduction to the Institute of Comparative Medicine (ICM), TC0800 The Mouse and Rat: Computer Based Training, and TC0550 Rodent Barrier Training, and attended the ICM wetlab taught by Columbia University veterinary staff. All animals were monitored daily by ICM staff and at minimum twice weekly by Kitajewski lab members. Mice were housed in an AAALAC-certified barrier environment with restricted access and autoclaved equipment. All mice for this study were purchased from Charles River or Jackson Labs. Mice were housed in cages of approximately 75 square inch floor space at no more than 5 adult female mice per cage, provided with bedding and nestlet enrichment. Bedding, water, and food were checked daily by ICM staff and changed weekly or more often as needed. When humane endpoints were met, euthanasia was performed by placing animals in a chamber, filling the chamber with CO_2_ at 30% of chamber volume per minute until at least 1 minute after cessation of respiration, and performing cervical dislocation to ensure death. Anesthesia was performed by administering 2–5% isoflurane using a precision vaporizer and nosecone, and sterile gel was applied to both eyes to prevent drying during the procedure.

2x10^6^ ID8 IP2 luciferase Notch3IC or Control tumor cells from Set #1 or Set #2 were suspended in phosphate buffered saline (PBS) and injected intraperitoneally (IP) into 6–8 week old female *Foxn1*^*nu*^ nude mice. IP injections are judged to cause only minimal and transient distress, so no anesthesia was used at this stage. A total of 76 mice were injected, 69 of which developed tumors. Tumor take failed in Notch3IC and Control injections at similar numbers. Mice were assessed weekly via circumference measurement and IVIS Spectrum In Vivo Imaging System (PerkinElmer) bioluminescent imaging. For IVIS imaging, mice were anesthetized, injected with 100μL of 30mg/mL luciferin (XenoLight D-Luciferin, Perkin Elmer), and imaged after 10 minutes. Circumference measurements were taken while mice were under anesthesia. Mice were sacrificed when they had reached 25% or greater increase in circumference or cachexia body condition score of BC1 or BC2 [35]. Six mice were found dead prior to reaching approved humane endpoint criteria. One cohort of mice was sacrificed at 8 weeks post-implantation regardless of tumor progression. At sacrifice, the peritoneal cavity was opened, peritoneal organs were removed, and the mice were imaged via IVIS to determine tumor burden in the peritoneal wall. The right ovary and attached uterine horn were dissected out together and IVIS imaged as well. 100 μL of 30mg/mL luciferin was mixed with 400 μL of phosphate buffered saline and directly added to tissue. Measurements were captured at 5 min post luciferin addition. ID8 IP2 cells were also injected into C57BL/6 hosts, but tumor onset was highly variable and luciferase expression was frequently lost in tumor cells.

### Statistics

All graphs and statistics were generated with Graphpad Prism 7 software using the statistical tests and sample sizes indicated in text and figure legends. T-test indicates two-tailed Welsh’s t-test with between-subject design and a significance threshold of p ≤ 0.05. Flow cytometry data (Fig 4C) was log_10_ transformed prior to t-test to account for the differences in magnitude of baseline ITGA1 expression between cell lines. Data from all samples and experiments were included with two exceptions: (a) data collected from cell line sets where post-facto Western blotting against Notch3IC showed poor lentiviral infection were excluded, and (b) mice injected with ID8 IP2 cells which did not develop tumors (i.e. no “tumor take”) were excluded. Failure of tumor take occurred in small numbers at similar rates between Control and Notch3IC injections. Outliers were not excluded. Mouse survival was analyzed with Mantel-Cox and Gehan-Breslow-Wilcoxon tests in Graphpad Prism versions 7 and 8. Correlation between Notch3 expression and human ovarian cancer was conducted using publicly available data at http://kmplot.com/analysis/index.php?p=service&cancer=ovar according to published algorithms [72]. KMplot data was analyzed using overall survival as a readout, excluding biased arrays, and restricted to serous ovarian cancer stages 1 and 2 for Fig 2B or serous ovarian cancer stages 3 and 4 for Fig 2C.

### Histology

Mouse tissue was dissected and was snap frozen in optimal cutting tissue compound (Tissue-Tek® O.C.T.). Hematoxylin and eosin (H&E) staining was performed on 5 μm frozen sections. Images were acquired on a Nikon Eclipse E800 Fluorescence Microscope with Nikon High-Resolution Digital Camera DXM 1200.

#### RNA sequencing

RNA was extracted from Notch3IC and Control Sets #1-#4 with a RNeasy Mini Kit (QIAGEN, 74104) and the RNase-Free DNase Set (QIAGEN, 79254). Library preparation and sequencing was performed by the JP Sulzberger Columbia Genome Center using Illumina TruSeq RNA Prep Kit with poly-A pulldown followed by sequencing ~30 million 100-base pair single-end reads on an Illumina HiSeq 2000 or 2500 instrument. Sequence was converted and demultiplexed using bcl2fastq, matched to the murine genome using TopHat, reads were determined with Cufflinks [73], and differential gene expression assessed with DESeq [74]. Multiple testing error was corrected using the Benjamini-Hochberg correction implemented in DEseq for adjusted p-values [75]. Genes with adjusted p-value < 0.1 and a Log_2_ fold change >|1| were subjected to pathway analysis using The Database for Annotation, Visualization and Integrated Discovery (DAVID) Bioinformatics Resources 6.7 analysis wizard (NIAID, NIH). Differentially expressed genes were converted to human orthologs using HomoloGene build 68 (NCBI, NIH) and analyzed with the Broad Institute’s Gene Set Enrichment Analysis (GSEA) [40]. The complete RNA sequencing dataset is available at accession GSE132737 in the NCBI Gene Expression Omnibus repository at https://www.ncbi.nlm.nih.gov/geo/query/acc.cgi?acc=GSE132737.

### Flow cytometry of Itga1

Mouse cells were dissociated from tissue culture plastic using a 1:4 mixture of Versene:Trypsin (0.05%) and human cells were dissociated using a 1:10 mixture of Cell dissociation buffer (Gibco 13150–016): Trypsin (0.05%). 5x10^6^ mouse or human cells infected with Notch3IC or Control overexpression vectors were stained with 0.2μg of either hamster anti-rat/mouse CD49a or isotype control conjugated to Alexa Flour 647 (BD Pharmingen 562113, and 562112) in 100μL of buffer (PBS with 0.1%NaN_3_ and 0.5% bovine serum albumin). Cells were washed, fixed with 1% paraformaldehyde in buffer, filtered through a 35μm nylon mesh, and analyzed on a Gallios flow cytometer **(**Beckman Coulter). FlowJo 10.2 was used for gating and analysis. The number of replicates for each experiment are indicated in each figure legend.

### Adhesion to extracellular matrix (ECM)

24-well plates were coated according to manufacturer specifications with Collagen I 5μg/cm^2^ (Corning 354236), collagen IV 10μg/cm^2^ (Corning 354233), fibronectin 5μg/cm^2^ (Corning 354008), laminin 10μg/cm^2^ (Corning 354232), or vitronectin 2μg/cm^2^ (R&D Systems 2349VN100) and blocked with 1% bovine serum albumin. 2x10^4^ cells were seeded of both Notch3IC and Control and incubated at 37°C in 5% CO_2_ for 30 to 90 minutes. Non-adherent cells were washed away with phosphate-buffered saline. Adherent cells were fixed with methanol and stained with 0.1% crystal violet. Cell were counted with a Celígo instrument and software (Nexcelom Bioscience). Sets #3–5 of ID8 IP2 were assessed in triplicate on 3 separate occasions. The Notch3IC mean was compared to the Control mean for each line.

### Migration scratch/wound assay

A confluent monolayer of cells in a 12-well plate was scraped with a p200 tip and the cells were allowed to close the gap. Photos were taken at 10x at 6 and 12 hours. Experiments were repeated in triplicate wells 3 times each for Sets #1, #2, and #4. Images processed in ImageJ (NIH) [76] with “find edges” and “sharpen” tools to increase contrast between the scratch and cellular areas and quantified with TScratch (Tobias Gebäck and Martin Schulz, ETH Zürich). For defined extracellular matrix, cells were seeded onto Oris collagen I or fibronectin coated 96 well plates (Platypus Technologies, CMACC1.101 or CMAFN1.101) and incubated overnight to create a confluent monolayer around the plug. Plugs were removed and cells were imaged at 0, 6, 12, and 24 hours and evaluated for confluence with a Celígo cytometer instrument and software (Nexcelom Biosciences). Sets #3-#5 were assessed in quadruplicate wells twice. Graphs display the average of the duplicate experiments for each set.

### Invasion

For Matrigel assessment, 1x10^4^ serum-starved cells were seeded in serum free media (DMEM 1% penicillin/streptomycin) onto BioCoat transwell inserts with 8μm pores pre-coated with Growth Factor Reduced Matrigel (Corning, 354483) or uncoated transwell inserts. Set #1 was evaluated in triplicate wells in 3 replicate experiments. Full-serum media (described above) was added below the insert. Cells were incubated for 24 hours, then fixed with methanol and stained with 0.1% crystal violet. Four 20x photos per insert were quantified by counting individual cells in ImageJ (NIH) [76].

Invasion through collagen I was assessed by seeding 2.5x10^5^ serum-starved cells onto CytoSelect 24-Well Cell Invasion Assay Collagen I, Colorimetric Format (Cell Biolabs, Inc., CBA-110-COL) in serum free media, and adding full-serum media to the well below the insert. Sets #3-#5 of ID8 IP2 were assessed in triplicate wells. After 72 hours, cells were stained, the stain was extracted according to manufacturer protocol and remaining stain was assessed at 560nm.

### Proliferation

Proliferation/ viability assays were performed by seeding 2500 cells per well in a 24 well plate and assessing the number of viable cells at 48 hours with WST-8 (Dojindo Molecular Technologies) absorbance readings at 450nm. 450nm readings were compared to readings of a standard curve generated from like cells. 5 separate experiments were performed with 3 replicates of both Notch3IC and Control. One matched set of lentiviral infected ID8 IP2 was assessed (Set #1). Graphs and statistical analysis were generated in GraphPad Prism 7.

### Colony formation in soft agar

Colony-forming plates were prepared by layering 0.75% agar in media, 0.75% agar and 2x10^4^ cells, 0.75% agar in media, and complete media in a 24 well plate in the listed order (see cell culture). Cells were incubated at 37°C in 5% CO_2_ for 3 weeks, changing media layer every other day. At 3 weeks, plates were stained with MTT at 37°C in 5% CO_2_ for 3 hours followed by washing with phosphate-buffered saline. Five 5X photos per well were taken and the number of colonies and area of colonies were evaluated with ImageJ (NIH) software. 4–6 wells were evaluated for each of 3 experiments conducted on one matched ID8 IP2 luciferase line set (Set #1). Graphs and statistics were generated with Graphpad Prism 7.

## Supporting information

S1 Fig Uncropped primary gel images. (A) Complete agarose gel images for semi-quantitative RT-PCR results used in Fig 1A. Lanes marked 1–6 are RNA samples from cell lines ID8 IP2, ID8 luc, OVCAR3, HUVEC, HBVP GFP, and water controls, respectively. Genes being tested are indicated under each set of samples. (B) Composite chemiluminescence and brightfield images for results in Fig 1B. (C) Composite chemiluminescence and brightfield images for results in Fig 1C. (D-E) Chemiluminescence images for Notch1 and Tubulin results in S4F Fig. (F-G) Chemiluminescence images for Notch3 and Actin results in S5A Fig.
(TIF)

S2 Fig ID8 IP2 ovarian cells cause disseminated tumors and ascites, features of human HGSC. (A) Mice intraperitoneally injected with ID8 IP2 cells exhibit ascites accumulation and (B) tumors that disseminate to sites throughout the peritoneal cavity, including the intestine, liver, and peritoneal wall (detail of boxed region of peritoneal wall in C). (D-E) H&E staining of two representative sections of an ID8 IP2 tumor, showing highly nucleated papillary tumors on the peritoneal wall.
(TIF)

S3 Fig Notch activation does not affect the survival of ID8 IP2 in vitro. (A) Notch target genes are robustly upregulated in each Notch3IC line compared to its matched Control, but qRT-PCR indicates variability in the magnitude of upregulation between lines. (B) ID8 IP2 Notch3IC show similar rates of viability/proliferation over a 48-hour period compared to Control. (C) ID8 IP2 Notch3IC do not form significantly more colonies than Control when grown in soft agar to assess anchorage independent growth.
(TIF)

S4 Fig Notch3IC display increased surface levels of ITGA1 by flow cytometry. (A-D) Representative gating strategy for flow cytometry. (A) Forward and side scatter gating to exclude dead cells and debris. (B) Negative control unstained ID8 IP2 parental cells. (C) Notch3IC cells stained with isotype control. The Notch3IC cells express GFP due to an IRES-GFP moiety of the Notch3IC construct. (D) Representative matched set of Control and Notch3IC cells stained with AF647-congugated anti-ITGA1 antibody. (E) ITGA1 surface expression is increased roughly 10 fold in Notch3IC cells compared to Control. Matched Sets #3–5 were assessed twice each, p = 0.0414, Welch’s t-test. The same data, averaged and transformed, is presented in Fig 4C, show here untransformed for easy comparison of fold changes. (F) Western blot of Notch1IC and Control cells, showing strong upregulation of Notch1IC protein. (G) ITGA surface expression is increased approximately 0.5 fold in Notch1IC cells compared to Control. Three independent matched sets were assessed once each, p = 0.0395, Welch’s t-test.
(TIF)

S5 Fig Increased Notch3 expression also upregulates ITGA1 in human ovarian cancer cells. (A) Representative Western blots show that expression of Notch3 intracellular domain is upregulated in Notch3IC lentivirally infected OVCA429 and OVSAHO cell lines. (B) qRT-PCR indicates that Notch3IC cells harbor significant upregulation of Notch 3 (p = 0.000001 for OVCA429 and p = 0.008691 for OVSAHO, Student’s t-test) and Hey L (p = 0.029 for OVCA429 and p = 0.013 for OVSAHO; error bars = S.E.M). (C) ITGA1 is upregulated by more than 10 fold on the surface of Notch3IC overexpressing cells as assessed by flow cytometry in a single experiment. (D-H) Representative gating strategy for flow cytometry for OVCA429 (top) and OVSAHO (bottom) cells. **(**D) Forward and side scatter gating to exclude dead cells and debris. (E) Unstained control cells. (F) Unstained N3ICD-expressing cells. (D-E) Representative matched sets of Control and Notch3IC overexpressing cells stained with AF647-congugated anti-ITGA1 antibody.
(TIF)

S1 Table Primers used for semi-quantitative RT-PCR and qRT-PCR for Notch receptors, Notch ligands, Notch3 downstream target genes, and control β-actin. (DOCX)

S2 Table Complete list of adhesion and extracellular matrix gene clusters. Determined by DAVID analysis to be significantly enriched in genes upregulated in Notch3IC cells, in order of ascending adjusted p value.
(DOCX)
