# Supplementary figures and images for: Notch3 signaling promotes tumor cell adhesion and progression in a murine epithelial ovarian cancer model
Source: PLoS One. 2020 Jun 11;15(6):e0233962. doi: 10.1371/journal.pone.0233962 (PMC7289394; doi:10.1371/journal.pone.0233962)

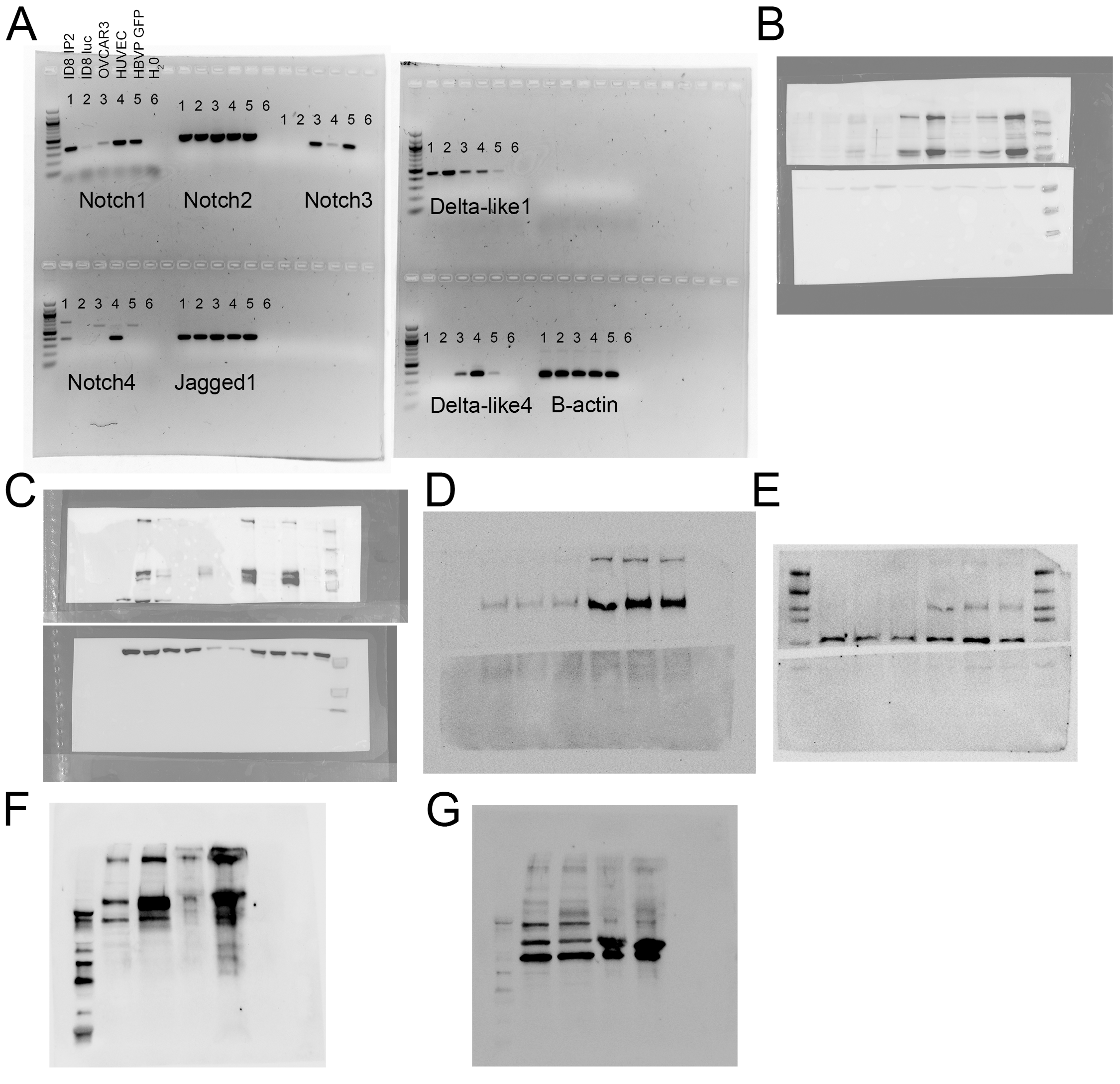

Supplement: S1 Fig — (A) Complete agarose gel images for semi-quantitative RT-PCR results used in Fig 1A. Lanes marked 1–6 are RNA samples from cell lines ID8 IP2, ID8 luc, OVCAR3, HUVEC, HBVP GFP, and water controls, respectively. Genes being tested are indicated under each set of samples. (B) Composite chemiluminescence and brightfield images for results in Fig 1B. (C) Composite chemiluminescence and brightfield images for results in Fig 1C. (D-E) Chemiluminescence images for Notch1 and Tubulin results in S4F Fig. (F-G) Chemiluminescence images for Notch3 and Actin results in S5A Fig. (TIF) [file pone.0233962.s001.tif]

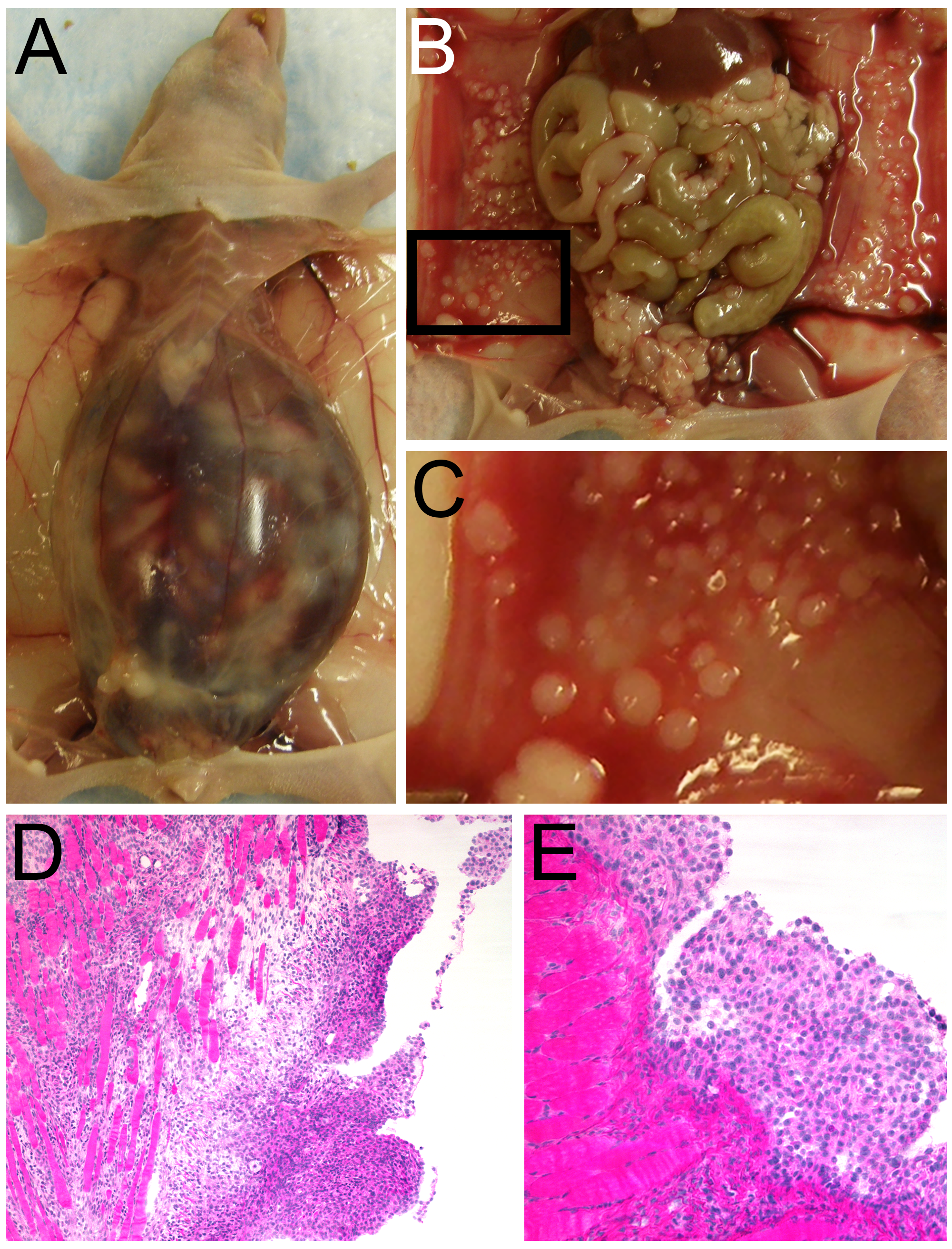

Supplement: S2 Fig — (A) Mice intraperitoneally injected with ID8 IP2 cells exhibit ascites accumulation and (B) tumors that disseminate to sites throughout the peritoneal cavity, including the intestine, liver, and peritoneal wall (detail of boxed region of peritoneal wall in C). (D-E) H&E staining of two representative sections of an ID8 IP2 tumor, showing highly nucleated papillary tumors on the peritoneal wall. (TIF) [file pone.0233962.s002.tif]

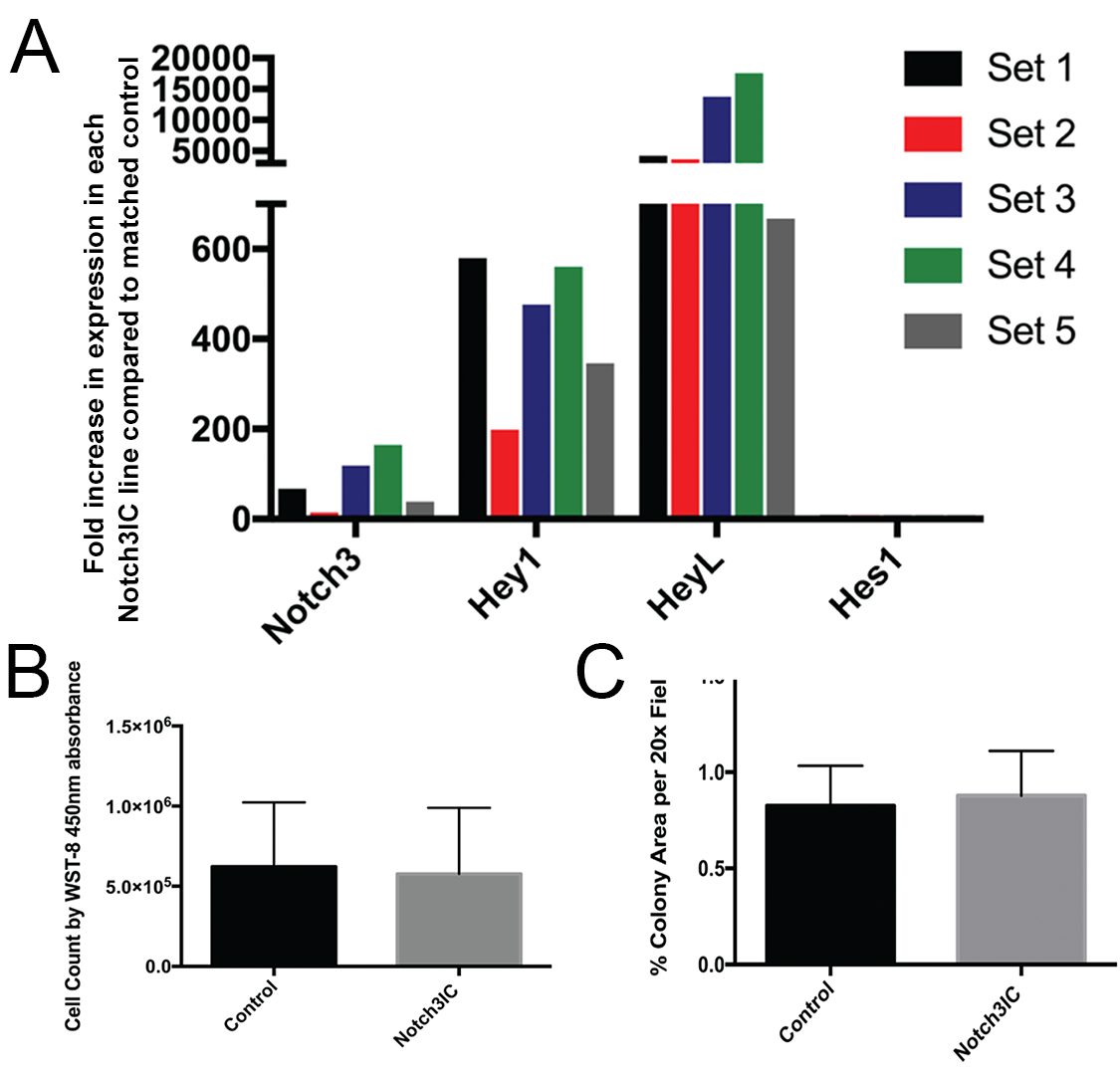

Supplement: S3 Fig — (A) Notch target genes are robustly upregulated in each Notch3IC line compared to its matched Control, but qRT-PCR indicates variability in the magnitude of upregulation between lines. (B) ID8 IP2 Notch3IC show similar rates of viability/proliferation over a 48-hour period compared to Control. (C) ID8 IP2 Notch3IC do not form significantly more colonies than Control when grown in soft agar to assess anchorage independent growth. (TIF) [file pone.0233962.s003.tif]

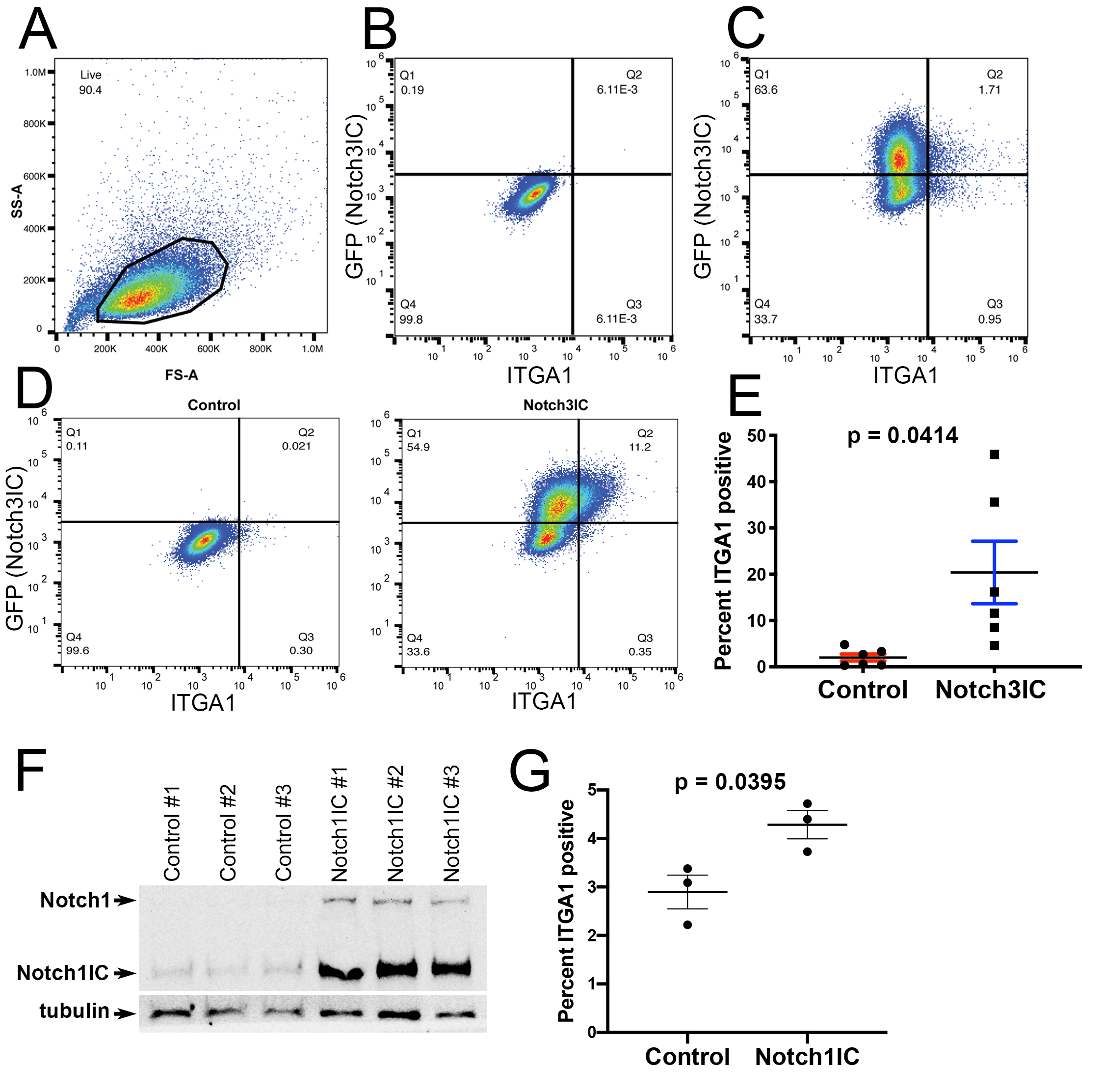

Supplement: S4 Fig — (A-D) Representative gating strategy for flow cytometry. (A) Forward and side scatter gating to exclude dead cells and debris. (B) Negative control unstained ID8 IP2 parental cells. (C) Notch3IC cells stained with isotype control. The Notch3IC cells express GFP due to an IRES-GFP moiety of the Notch3IC construct. (D) Representative matched set of Control and Notch3IC cells stained with AF647-congugated anti-ITGA1 antibody. (E) ITGA1 surface expression is increased roughly 10 fold in Notch3IC cells compared to Control. Matched Sets #3–5 were assessed twice each, p = 0.0414, Welch’s t-test. The same data, averaged and transformed, is presented in Fig 4C, show here untransformed for easy comparison of fold changes. (F) Western blot of Notch1IC and Control cells, showing strong upregulation of Notch1IC protein. (G) ITGA surface expression is increased approximately 0.5 fold in Notch1IC cells compared to Control. Three independent matched sets were assessed once each, p = 0.0395, Welch’s t-test. (TIF) [file pone.0233962.s004.tif]

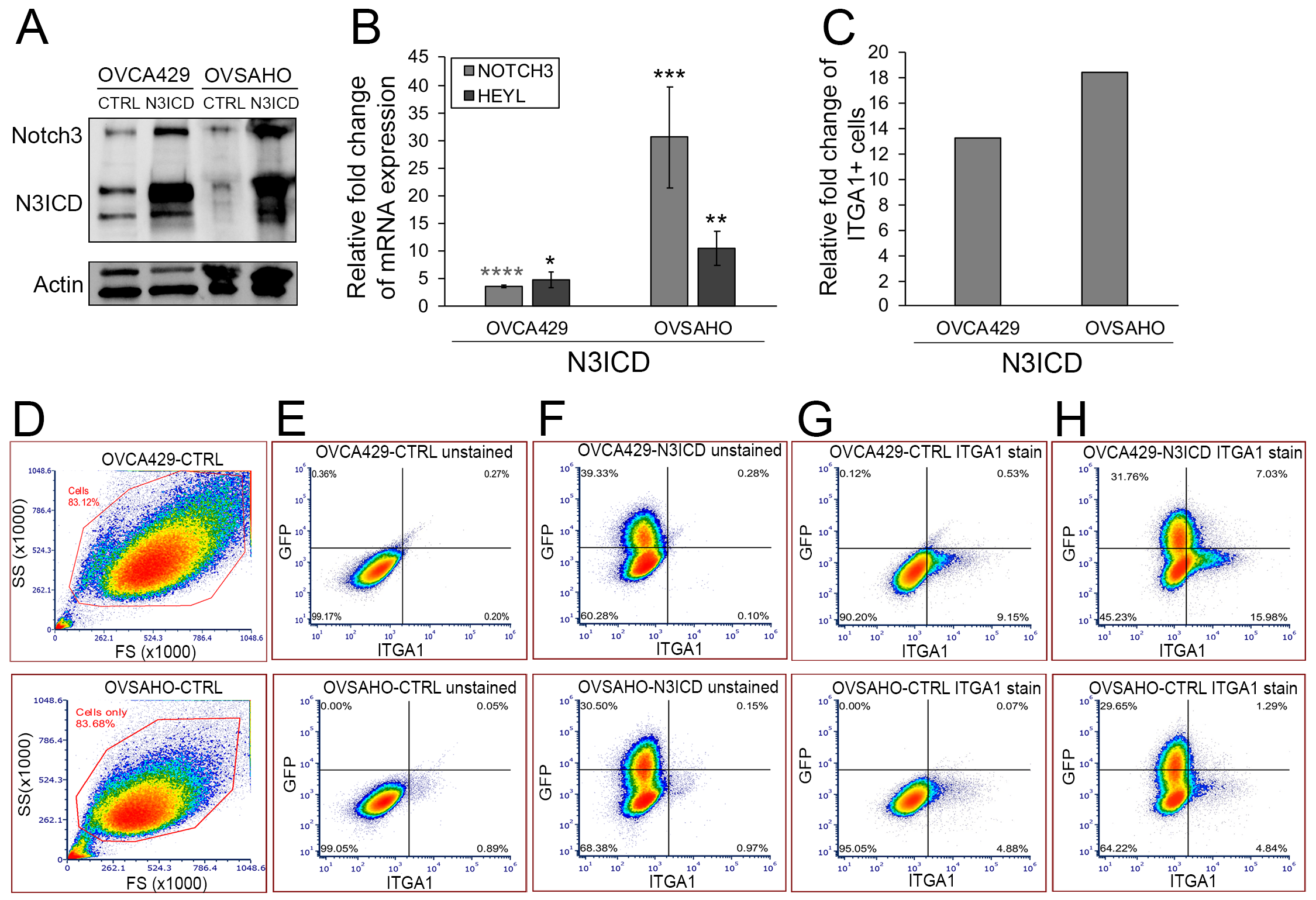

Supplement: S5 Fig — (A) Representative Western blots show that expression of Notch3 intracellular domain is upregulated in Notch3IC lentivirally infected OVCA429 and OVSAHO cell lines. (B) qRT-PCR indicates that Notch3IC cells harbor significant upregulation of Notch 3 (p = 0.000001 for OVCA429 and p = 0.008691 for OVSAHO, Student’s t-test) and Hey L (p = 0.029 for OVCA429 and p = 0.013 for OVSAHO; error bars = S.E.M). (C) ITGA1 is upregulated by more than 10 fold on the surface of Notch3IC overexpressing cells as assessed by flow cytometry in a single experiment. (D-H) Representative gating strategy for flow cytometry for OVCA429 (top) and OVSAHO (bottom) cells. (D) Forward and side scatter gating to exclude dead cells and debris. (E) Unstained control cells. (F) Unstained N3ICD-expressing cells. (D-E) Representative matched sets of Control and Notch3IC overexpressing cells stained with AF647-congugated anti-ITGA1 antibody. (TIF) [file pone.0233962.s005.tif]
