# Supplementary material for: Notch3 signaling promotes tumor cell adhesion and progression in a murine epithelial ovarian cancer model
Source: PLoS One. 2020 Jun 11;15(6):e0233962. doi: 10.1371/journal.pone.0233962 (PMC7289394; doi:10.1371/journal.pone.0233962)
Supplement: S1 Table — (DOCX) [file pone.0233962.s006.docx]

**Table S1**. **Primers used for semi-quantitative RT-PCR and qRT-PCR for Notch receptors, Notch ligands, Notch3 downstream target genes, and control β-actin.**
