# Supplementary material for: Notch3 signaling promotes tumor cell adhesion and progression in a murine epithelial ovarian cancer model
Source: PLoS One. 2020 Jun 11;15(6):e0233962. doi: 10.1371/journal.pone.0233962 (PMC7289394; doi:10.1371/journal.pone.0233962)
Supplement: S2 Table — Determined by DAVID analysis to be significantly enriched in genes upregulated in Notch3IC cells, in order of ascending adjusted p value. (DOCX) [file pone.0233962.s007.docx]

**Table S2. Complete list of adhesion and extracellular matrix gene clusters.** Determined by DAVID analysis to be significantly enriched in genes upregulated in Notch3IC cells, in order of ascending adjusted p value.
